# Validation of Suitable Reference Genes for Gene Expression Studies on Yak Testis Development

**DOI:** 10.3390/ani10020182

**Published:** 2020-01-21

**Authors:** Xuelan Zhou, Xiaoyun Wu, Min Chu, Chunnian Liang, Xuezhi Ding, Jie Pei, Lin Xiong, Pengjia Bao, Xian Guo, Ping Yan

**Affiliations:** Key Laboratory of Yak Breeding Engineering, Lanzhou Institute of Husbandry and Pharmaceutical Sciences, Chinese Academy of Agricultural Sciences, Lanzhou 730050, China; zhouxl17@lzu.edu.cn (X.Z.); wuxiaoyun@caas.cn (X.W.); chumin@caas.cn (M.C.); liangchunnian@caas.cn (C.L.); dingxuezhi@caas.cn (X.D.); peijie@caas.cn (J.P.); xionglin@caas.cn (L.X.); baopengjia@caas.cn (P.B.)

**Keywords:** expression stability, normalization, quantitative real-time polymerase chain reaction, RNA

## Abstract

**Simple Summary:**

Yak (*Bos grunniens*) provides life materials for herdsmen in high-plateau areas. Improving their low fertility is necessary to meet the demands of the development of the yak industry. The testis is an important organ for male fertility, its development is controlled by a large number of genes. Using real-time reverse transcriptase-quantitative polymerase chain reaction (RT-qPCR) to explore the quantitative expression of genes can provide insights for illuminating the molecular mechanisms of testis development, but the RT-qPCR data are influenced by the stability of reference genes (RGs). Unfortunately, no available RGs can normalize the gene expression in yak testis development. In this study, the expression stability of 13 candidate genes in yak testis at different developmental stages was evaluated using five different pieces of software. The results showed that the TATA box-binding protein (*TBP*) and ubiquitously expressed transcript protein (*UXT*) exhibited high stability across various developmental stages, *TBP* and hydroxymethylbilane synthase (*HMBS*) were the most stably expressed genes in immature stages, and mitochondrial ribosomal protein L39 (*MRPL39*) and *TBP* exhibited the most stable expression across mature stages. This study provided suitable RGs for gene expression studies in yak testis development.

**Abstract:**

Testis has an important function in male reproduction. Its development is regulated by a large number of genes. The real-time reserve transcriptase-quantitative polymerase chain reaction (RT-qPCR) is a useful tool to evaluate the gene expression levels. However, unsuitable reference genes (RGs) can cause the misinterpretation of gene expression levels. Unfortunately, the ideal RGs for yak testis development are yet to be studied. In this study, 13 commonly used RGs were selected to identify the most stable RGs in yak testis at four different developmental stages, including two immature stages (6 months and 18 months) and two mature stages (30 months and 6 years). This study used GeNorm, NormFinder, BestKeeper, ∆*Ct*, and RefFinder programs to evaluate the stability of 13 candidate genes. The results of RefFinder showed that the stabilities of TATA box-binding protein (*TBP*) and ubiquitously expressed transcript protein (*UXT*) were ranked the top two across all developmental stages. *TBP* and hydroxymethylbilane synthase (*HMBS*) were stably expressed in immature stages, while mitochondrial ribosomal protein L39 (*MRPL39*) and *TBP* had higher stability than other candidate genes in mature stages. This study provided valuable information for gene expression studies to assist further investigation on the molecular mechanisms in underlying yak testis development.

## 1. Introduction

Yak (*Bos grunniens*) is mainly distributed in the Qinghai-Tibetan plateau. As the most important domestic animal in this area, it can feed on poor-quality roughage, adapt to harsh environments, and provide many products, such as meat, milk, and leather, for local people [1]. Despite these merits, yaks exhibit lower reproductive performance, including delayed onset of puberty, seasonal reproductive patterns, low conception rate, and long calving intervals [2]. Lower reproductive efficiency has severely restricted the progress in breeding and development of the yak industry.

The testis has an important function in male reproduction. It can secrete several male steroids hormones and continuously produce sperms. Spermatogenesis is an exceedingly complex process and crucial for male fertility. It includes three specific functional phases: Mitotic proliferation of spermatogonia, meiosis of spermatocytes, and spermiogenesis of haploid spermatids [3]. The previous study found that with increasing age, the testis of yak significantly increased in weight, the tunica albuginea became continuously thicker, and the surface blood vessels became larger and denser. A histological observation of yak testis indicated that testicular seminiferous tubules only contained spermatogonia and Sertoli cells at the age of three months, and sperms could be detected in the epididymis at the age of 18 months Reproductive maturity marker (lactate dehydrogenase) appeared in yak testis at two years of age, indicating that yak bulls reached physical maturity and produced matured sperm at this age [4]. Previous studies showed that some key genes involved in male reproduction and spermatogenesis process were differentially expressed at different developmental stages [5,6,7,8]. To further understand the mechanism underlying yak testis development, the details of the expression patterns of genes related to male fertility need to be further verified by real-time reverse transcriptase-quantitative polymerase chain reaction (RT-qPCR).

The RT-qPCR approach has some advantages, such as high throughput, accuracy, sensitivity, and reproducibility. Therefore, it is widely used for measuring and evaluating gene expression levels [9]. However, a few variables can easily influence the quantification results of RT-qPCR, such as RNA integrity and quantity, accurate reverse transcription, and primer efficiency. However, the RT-qPCR data bias can be minimized using stably expressed genes as RGs to calibrate the expression levels [10]. Ideal RGs should be stably expressed across different developmental stages and types of tissues [11]. Unsuitable RGs can cause misjudgment in the quantitation of genes and even produce false results [12]. A set of appropriate RGs is essential for gene normalization. Suitable RGs of yak species have been identified in mammary tissue and milk somatic cells during the lactation cycle, and different tissues of fetal yak [13,14,15]. However, the ideal RGs in yak testis development have still not been validated. In this study, 13 commonly used candidate RGs were selected to identify a set of suitable RGs in yak testis at different developmental stages. The expression stabilities of candidate RGs in testis at different developmental stages were assessed using five different algorithms (geNorm [16], NormFinder [17], BestKeeper [18], ∆*Ct* [19], and RefFinder [20]). Finally, the selected RGs were used to normalize the expression of the Tet methyl cytosine dioxygenase 2 (*TET2*) gene in yak testis at different developmental stages.

## 2. Materials and Methods

### 2.1. Animals and Sample Collection

A total of 12 healthy male Datong yaks used in this study were raised at the Qinghai Datong yak farm (Qinghai, China) in the same environment. Samples of the testis in the immature stages (6 months old and 18 months old) and mature stages (30 months old and 6 years old) were collected immediately after slaughter. The mature male yaks were used for natural breeding. Three different yaks of each age were sacrificed for sample collection. All samples were collected in a microtube (1.5 mL), immediately frozen in liquid nitrogen, and stored at −80 °C in a refrigerator until further use. All the experimental protocols and procedures were approved by the Animal Administration and Ethics Committee of Lanzhou Institute of Husbandry and Pharmaceutical Sciences of CAAS (Permit No. SYXK-2014-0002).

### 2.2. RNA Isolation and cDNA Synthesis

Total RNA was isolated from testis using the animal tissue RNA isolation kit (ZDGSY, Beijing, China). The purity and concentration of total RNA were confirmed using the NanoDrop2000 spectrophotometer (Thermo Fisher Scientific, Waltham, MA, USA). The first-strand cDNA was synthesized from 1000 ng total RNA using the PrimeScript RT reagent kit with gDNA Eraser (TaKaRa, Dalian, China) in a total of 20 µL of the reaction mixture.

### 2.3. Selection of Candidate RGs and Primer Design

To identify suitable RGs in yak testis at different developmental stages, 13 candidate RGs (*TBP*, *ACTB*, *PPIA*, *HPRT1*, *GAPDH*, *SDHA*, *UXT*, *YWHAZ*, *RPL13A*, *RPS15*, *HMBS*, *MRPL39,* and *PPP1R111*) which are commonly used as appropriate RGs, were selected for RT-qPCR assays according to the literature report (Table 1). Among these candidate RGs, *PPP1R11*, *UXT*, *MRPL39*, *RPS15*, *HMBS*, *YWHAZ*, *TBP,* and *ACTB* have been reported to be optimal RGs for normalization in yak [13,14,15,21]. In addition, *GAPDH*, *HPRT1*, *PPIA*, *SDHA,* and *RPL13A* were the commonly used RGs for expression studies in testis tissue and primordial germ cells of mice and rats [22,23,24,25]. Primers for *TBP*, *ACTB*, *PPIA*, *HPRT1*, *GAPDH*, and *SDHA* were reported by Li et al. [14]. *UXT*, *YWHAZ*, *RPL13A*, *RPS15*, *HMBS*, *MRPL39,* and *PPP1R11* were designed based on the sequence obtained from the National Center for Biotechnology Information using Primer Premier 5.0 software. The size of the RT-qPCR products was between 79 and 190 bp.

### 2.4. RT-qPCR Assay

The generated cDNAs were diluted tenfold in RNase-free water. The RT-qPCR was carried out on a Bio-Rad CFX96 Real-Time PCR System (Bio-Rad Laboratories, Hercules, CA, USA) using 96-well plates. The total volume of each reaction mixture contained 10 µL of SYBR TB Green mix (TaKaRa), 1 µL of each primer, 1 µL of diluted cDNA, and 7 µL of RNase-free water. The procedure was conducted as follows: 95 °C for 3 min, followed by 45 cycles of 95 °C for 10 s, 60 °C for 10 s, and 72 °C for 10 s. The melting curve was obtained by melting the amplicon with constant heating from 65 °C 5 s to 95 °C. The specificity of RT-qPCR products was judged by melting curves. Each reaction was run in triplicate. The standard curves were generated to calculate the reaction efficiency and the correlation coefficient (*R^2^*). The five-point standard curve was generated using a tenfold dilution series of cDNA samples. The coefficient of determination (*R^2^*) and slope were calculated from the linear regression model, and the mean amplification efficiency (*E*) of each primer pair was calculated using the following formula: *E* (%) = [10^(−1/slope)^ − 1] × 100 [26].

### 2.5. Stability Analysis of RGs

The expression stability of the 13 selected RGs was evaluated using four programs: geNorm [16], Normfinder [17], Bestkeeper [18], Delta Ct [19] and a comprehensive online tool RefFinder [20] (http://150.216.56.64/referencegene.php) according to the developer’s instructions. GeNorm was also used to calculate the pairwise variation (*V*_n_/*V*_n+1_ value) and determine the minimum number of RGs required for accurate normalization.

### 2.6. Validation of Expression of RGs

The stabilities of the identified best RGs were validated by measuring the expression level of the *TET2* gene in the different tested groups. The gene expression of *TET2* was normalized using the two most stable candidate RGs and the combination of two most stable RGs, as well as the least stable RG. The following experimental run protocol was used with an initial denaturation at 95 °C for 30 s, and 45 cycles of 95 °C for 5 s and 60 °C for 30 s. The relative expression levels of *TET2* mRNA were analyzed using the 2^−ΔΔCt^ method [27].

## 3. Results

### 3.1. Primer Specificity, Amplification Efficiency, and Gene Expression Profiles

A total of 13 candidate RGs were selected to identify the possible RGs for RT-qPCR analyses in yak testis at different developmental stages. The dissociation curves of the products presented a single peak, indicating a specific melting temperature (Figure S1). Among all the primers, the efficiency ranged from 92.0% to 108.0%, and the *R^2^* values were greater than 0.99 (Table S1).

### 3.2. Ct Value

**Figure 1:**
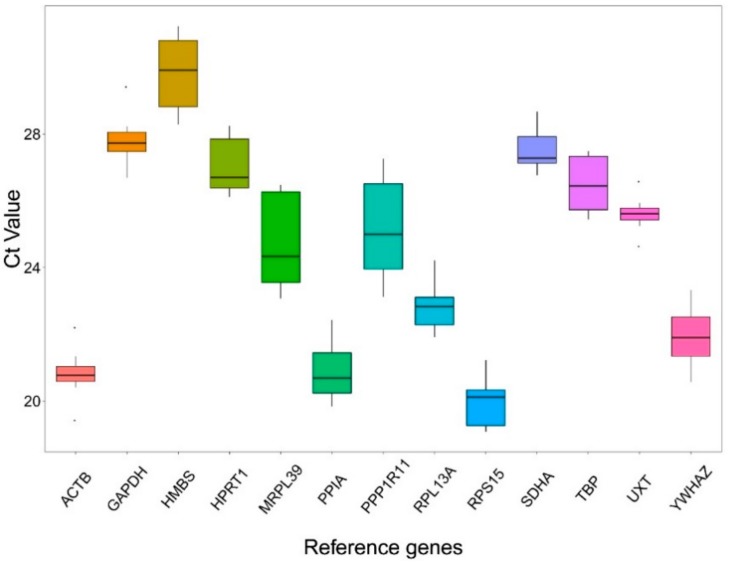
Ct values of 13 candidate RGs across all tested samples. The lower and upper box represents the 25th and 75th percentiles and the whiskers caps represent the maximum and minimum values. A center line across the boxes indicates the median.

The Ct values in RT-qPCR provided an overview of the expression levels of 13 candidate RGs in 12 samples (Figure 1). Obvious differences in expression abundance among genes were found. A lower Ct value represented a higher expression level. Conversely, a higher Ct value represented a lower expression level. Among the 13 analyzed genes, *RPS15* had the highest expression level, while *HMBS* was the least abundantly expressed gene with the highest Ct value. *ACTB* and *UXT* displayed the lowest expression variations across all the samples. On the contrary, *MRPL39* and *PPP1R11* exhibited the highest variations. Descriptive statistics of the Ct values are presented in Table S2.

### 3.3. Expression Analysis of Candidate RGs in Yak Testis at Different Developmental Stages

Comprehensive expression analysis of candidate RGs was conducted for different developmental stages. Statistical analyses were performed in the overall dataset comprising all samples as well as in two subsets (sample groups) distinguished due to the different stages. The stability rankings of the candidate RGs using the four programs (geNorm, Normfinder, BestKeeper, and Delta Ct) were varied, as well as the stability order of the RGs at different developmental stages varied (Table 2, Table 3 and Table 4).

#### 3.3.1. GeNorm Analysis of RGs

**Figure 2:**
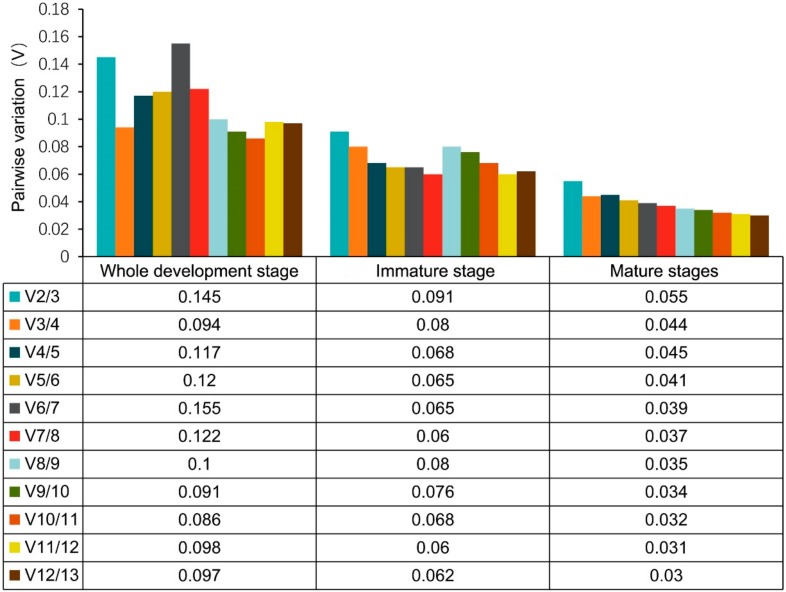
Pairwise variation (V) analyses of the candidate RGs in immature, mature, and whole development stage. GeNorm program calculates the pairwise variation (Vn/n + 1) to determine the effect of additional RG on accurate normalization. The cutoff threshold was proposed to be 0.15, below which the inclusion of an additional RG is not required.

The geNorm algorithm calculates the M-values to determine the stability in gene expression. The stability in expression for the 13 candidate genes was ranked according to the M-value, and the most stably expressed genes showed the lowest M-value. A gene with M value >1.5 was regarded as the unacceptable level of expression variability. During all developmental stages in this study, each candidate RG exhibited relative stability with the threshold value below 1.5. When all developmental stages were analyzed as one data set, the results showed that *RPS15* and *RPL13A* were the most stable RGs with the lowest M value of 0.153, whereas *PPP1R11* with the highest M value of 1.033 was the least stably expressed gene. In the sexually immature stage set, *TBP* and *HMBS* (M = 0.191), was assessed as the best-ranked RGs, while *PPIA* (M = 0.697) showed the most variable expression. For the samples of the sexually mature stage, *PPIA* and *MRPL39* (M = 0.139) were considered as the most stable genes, whereas *SDHA* was the least stable gene (M = 0.139). The geNorm analysis showed that the most stable genes were variable at different developmental stages (Table 2, Table 3 and Table 4). As shown in Figure 2, all pairwise variations values of V_2_/V_3_ for RGs were less than 0.15, indicating that a combination of two RGs could be used for the accurate normalization of gene expression at each developmental stage in this study.

#### 3.3.2. NormFinder Analysis of Expression Stability of RGs

According to the NormFinder program, genes with the lowest stability values were the most stably expressed. For all developmental stages, *TBP* (0.345) and *YWHAZ* (0.46) were the most stably expressed genes, while *PPP1R11* (1.259) showed the highest variation. For the samples at the immature stage, *TBP* and *HMBS* were regarded as the top two stably expressed RGs with the lowest stability values of 0.258 and 0.287, respectively, whereas *PPIA* exhibited the lowest stability with the highest stability value of 0.807. During the mature stage, *TBP* (0.102) had the most stable expression with the lowest stability value, followed by *MRPL39* (0.129), while *SDHA* (0.383) had the highest stability value (Table 2, Table 3 and Table 4). *TBP* was the most stable gene (with the lowest stability value) in all data subsets, which could be used as the most stable RG for the normalization of target gene expression in yak testis at different developmental stages.

#### 3.3.3. BestKeeper Analysis of Expression Stability of RGs

The BestKeeper software was used to evaluate the expression stability of candidate RGs based on their standard deviation (SD). Genes with the lowest SD value were considered as acceptable RGs. As shown in Table 2, *UXT* (0.31) and *ACTB* (0.43) were the top two stable RGs at all developmental stages and immature stages. For the mature stage samples, *HPRT1*(0.16) and *PPIA* (0.28) were considered as the top two stable RGs. *PPP1R11* (1.36), *PPIA* (0.78), and *SDHA* (0.56) were ranked as least stable RGs at all developmental stage samples, immature stage samples, and maturity stage samples, respectively (Table 2, Table 3 and Table 4).

#### 3.3.4. ∆Ct Method Analysis of Expression Stability of RGs

Similar to the other three programs, genes with the lowest stability values were ordered as the most suitable RGs. The ∆*Ct* method results are shown in Table 2. *TBP* (0.82) and *YWHAZ* (0.87) were identified as the top two suitable RGs, while *PPP1R11* (1.35) exhibited the lowest stability in all samples. On the contrary, *TBP* (0.55) and *HMBS* (0.56) were recommended as the top two suitable RGs in the immature stage, and *PPIA* (0.93) was ranked as the least stable RG. *TBP* (0.29) and *MRPL39* (0.29) showed high stability in the mature stage, whereas *SDHA* (0.32) was variably expressed in this stage (Table 2, Table 3 and Table 4). *TBP* was the most stable gene at all developmental stages, similar to the NormFinder results.

#### 3.3.5. RefFinder Ranking of the Most Stable Genes

The RefFinder is a comprehensive online tool to determine the ranking of stability of RGs according to the geometric mean of the ranking, which was calculated using the geNorm, NormFinder, BestKeeper, and ∆*Ct* algorithms in this study. Candidate genes with the lowest geometric mean were the most stable genes. According to comprehensive ranking results, *TBP* (2.74) and *UXT* (3.46) with the lowest values were identified as the most suitable RGs at all developmental stage samples. *TBP* and *HMBS* were recommended as the top two stably expressed genes with low Ct values of 1.57 and 2.45, respectively, in the immaturity stage. On the contrary, the most stably expressed genes were *MRPL39* (2.11) and *TBP* (2.63) in the mature stage samples. *PPP1R11* (13.00), *PPIA* (13.00), and *SDHA* (13.00) with high values were unsuitable for RGs in all stage samples, immature stage samples, and mature stage samples, respectively (Table 2, Table 3 and Table 4).

### 3.4. Validation of Candidate RGs

**Figure 3:**
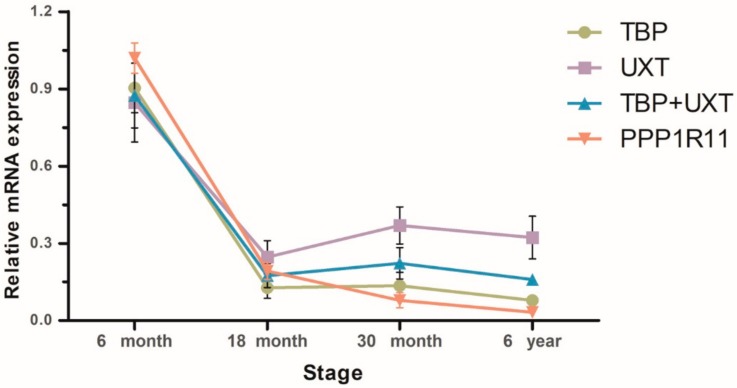
The expression pattern of the *TET2* mRNA in the whole developmental stage of yak testis. The expression patterns were normalized using *TBP*, *UXT,* or *PPP1R11* as RGs singly and with the mean of *TBP* + *UXT*.

**Figure 4:**
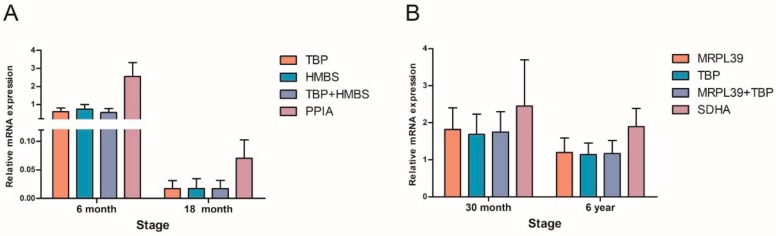
Relative expression levels of *TET2* at the immature stage and mature stage normalized by the selected stable RGs (singly or in combination) and the least stable gene. (**A**) Immature stage, (**B**) mature stage.

To validate the influences of different RGs on gene expression levels, the expression patterns of *TET2* in yak testis were detected at different developmental stages. It was observed that the mRNA expression trends of *TET2* were similar when using the two most stable RGs either singly or in combination, while different expression trends were obtained when using the least stable RG (Figure 3). In the immature and mature stages, the expression patterns of *TET2* were fully overestimated when using the least stable RGs (Figure 4). Unsuitable RGs may cause misinterpretation of the expression of target genes. Therefore, it is essential to use the appropriate stable RGs for relative gene expression.

## 4. Discussion

The testis development is an important and strictly regulated process in mammals, which needs precise regulation of a great number of genes and networks [5,28,29]. Unraveling the molecular mechanisms underlying testis development may benefit the effective utilization in animal breeding. Expression analysis of fertility-related genes by RT-qPCR is an important approach for revealing the molecular mechanism underlying testis development. The expression results of target genes can be interpreted accurately and reliably by selecting a set of suitable RGs under any experimental condition. In this study, 13 candidate RGs were selected, which were widely used in gene relative quantification analysis to evaluate the expression stability of RGs.

The ideal RG should be stable throughout the physiological state and with minor variations during experimental conditions. However, no universal RG exists that can be used under all experimental conditions [30]. For example, *GAPDH* is one of the commonly used RGs to evaluate the expression levels of target genes in the testis development process of some species [31,32]. However, studies have shown that *GAPDH* is not the best option, and its expression levels may be influenced by the samples from different tissues or different developmental stages, as well as the experimental conditions [33]. Bioinformatics analyses showed that *GAPDH* had many pseudogenes in the human and mouse genome, and the fidelity of RT-qPCR data might be affected by the specificity of primer design [34]. In this study, *GAPDH* was also not a suitable gene for normalization in yak testis development, which was consistent with the result of a previous study [35]. However, Gong et al. identified *GAPDH* as one of the suitable RGs in mouse testis development [25]. These studies suggested that choosing RGs only from the literature reports was not sufficient. Selecting the suitable RGs for normalization is essential before investigating the expression levels of target genes under specific experimental conditions.

In this study, four widespread algorithms (geNorm, NormFinder, BestKeeper, and Δ*Ct*) were employed to validate the most stably expressed genes in different subsets. Similar to other studies, this study also showed that the most stable and least stable RGs were ranked variably using different algorithms, which might be attributed to distinct statistical algorithm procedures [16,17,18,19]. Although the ranks were similar in some algorithms, no fixed standard existed. Therefore, a comprehensive algorithm was needed that would integrate the aforementioned four algorithms to rank gene stability. A previous study showed that the conventional use of a single gene for normalization resulted in relatively large errors in a significant percentage of the tested samples [21]. Currently, many studies used multiple RGs for normalization to obtain more accurate and reliable data [36]. In this study, geNorm analysis showed the values of V_2_/V_3_ in each stage of testis development to be less than 0.15, indicating that only two most stable RGs were sufficient for gene expression normalization at each testis developmental stage.

Based on the RefFinder results, *TBP* combined with *UXT* was the preferred combination of stable RGs for all developmental stages, *TBP* combined with *HMBS* was recommended as the best RG combination for immature stages, and *MRPL39* and *TBP* were identified as the most stable genes for mature stages. *TBP* showed high stability at different developmental stages of yak testis. *TBP* is an essential transcription factor, which belongs to a small gene family of TBP-related factors, and plays a role in the activation of eukaryotic genes transcribed by RNA polymerase II [37]. *TBP* is identified as the most stable gene in fetal mouse gonads, and in human fetal testis [38,39]. The results of this study were consistent with those of previous studies, which showed that *TBP* was considered as the most stable RG in porcine testis development [40]. However, *TBP* is regarded as the least stable gene in mouse testis development [25]. These results indicated that the expression stability of *TBP* in the testis development process might be affected by species. *UXT* has an important role in normal centrosomal biogenesis and cell survival [41]. *MRPL39* is a nuclear gene coding for a constituent of mitochondrial ribosomes, which are essential for the mitochondrial protein synthesis [42]. *UXT* and *MRPL39* are also two of three best internal control genes in the mammary tissue of yak during the lactation cycle [13], indicating that these two genes were the suitable RGs in yak species. *HMBS* was considered as the stably expressed gene in immature stages, which was similar to previous findings on the spermatozoa of buffalo during freezing and thawing [43]. Therefore, these genes could be selected as the most appropriate pair of RGs in yak testis development. A previous study demonstrated that *PPP1R11* was the most stable gene in milk somatic cells of yak [15], while the present study reported contradictory results. The results of this study regarded *PPP1R11* as the unstable RG at all developmental stages. These results indicated that the expression of *PPP1R11* was influenced by the types of yak tissues. A series of studies showed that the ideal RGs might vary under different experimental conditions, even in the same species. Therefore, RGs should be validated in various experiments.

DNA methylation is critical for mammalian animal testis development [44]. *TET2* is a crucial regulator of DNA methylation. It has been identified as a key enzyme for catalyzing the conversion of methyl cytosine into 5-hydroxymethylcytosine. Spermatogenesis is a complicated cell differentiation process accompanied by a drastic epigenetic change [45]. A recent study showed that *TET2* mRNA was successively expressed during human spermatogenesis, and its expression levels were associated with male fertility [46]. In this study, the expression levels and tendency of *TET2* were normalized by the two most stable genes and one least stable gene, respectively. The relative expression pattern of *TET2* showed a strong deviation when normalization was performed using the unstable RG. Hence, an unsuitable RG may mislead the research orientation. Therefore, assessing the stability of RGs under different experimental conditions is necessary.

## 5. Conclusions

The results showed that *TBP* combined with *UXT* was the preferred combination of stable RGs in yak testis at all developmental stages. *TBP* combined with *HMBS* was recommended as the best RG combination for immature stages, and *MRPL39* and *TBP* were identified as the most stable genes for the mature stages. The set of suggested reliable RGs is specific only for the present experiment and can be used as such if the same experiment is performed, such as the same yak breed and time points of testis development. If anything changes in the experimental model, the proposed RGs should be tested to assess their reliability under different experimental conditions.

## Figures and Tables

**Table 1:** Gene-specific primers for candidate RGs.

Gene	Gene Name	Accession No.	Primer Sequence (5′-3′)	Size (bp)
*ACTB*	β-actin	XM_005887322.2	F: ATTGCCGATGGTGATGAC R: ACGGAGCGTGGCTACAG	177
*GAPDH*	glyceraldehyde 3 phosphate dehydrogenase	XM_014482068.1	F: TCACCAGGGCTGCTTTTA R: CTGTGCCGTTGAACTTGC	126
*UXT*	Ubiquitously Expressed Prefoldin Like Chaperone	XM_005899362.2	F: AGGTGGATTTGGGCTGTAAC R: CTTGGTGAGGTTGTCGCTGA	170
*TBP*	TATA box-binding protein	NW_005395834.1	F: GTCCAATGATGCCTTACGG R: TGCTGCTCCTCCAGAATAGA	82
*YWHAZ*	Tyrosine 3-Monooxygenase/Tryptophan 5-Monooxygenase Activation Protein Zeta	XM_005887010.2	F: AATGTTGTAGGAGCCCGTAG R: CTGCTTGTGAAGCGTTGG	190
*RPL13A*	Ribosomal Protein L13a	XM_014481217.1	F: CAAGCGGATGAACACCAA R: GCAGCAGGAACCACCATT	192
*SDHA*	Succinate Dehydrogenase Complex Flavoprotein Subunit A	XM_005894659.2	F: GGGAACATGGAGGAGGACA R: CCAAAGGCACGCTGGTAGA	188
*RPS15*	Ribosomal Protein S15	XM_005890466.2	F: GACCTTCCGCAAGTTCACCT R: ACCACCTCGGGCTTCTCCAT	198
*HPRT1*	hypoxanthine guanine phosphoribosyl transferase 1	XM_005911180.2	F: GTGATGAAGGAGATGGG R: ACAGGTCGGCAAAGAAC	79
*PPIA*	peptidylprolyl isomerase A	XM_005891872.2	F: TTTTGAAGCATACAGGTCC R: CCACTCAGTCTTGGCAGT	98
*HMBS*	Hydroxymethylbilane Synthase	XM_005897126.2	F: GAACAAAGGAGCCAAGAAC R: CAGAGGGCTGGGATGTAG	121
*MRPL39*	Mitochondrial Ribosomal Protein L39	XM_005898618.2	F: AAACCTTTGACCAAGTCCTGT R: TTCCTCTTTGAATGCCCTCTC	135
*PPP1R11*	Protein Phosphatase 1 Regulatory Inhibitor Subunit 11	XM_005911410.2	F: CAGAAAAGACAGAAGGGTGC R: TTCCGAAGTTTGATGGTTAG	164
*TET2*	Tet methyl cytosine dioxygenase 2	XM_005890479.1	F: ATGAAAGGAAGCCAAAAGAG R: ATGGAGCCCAGAGAGAGAAG	126

**Table 2:** Stability of RGs all developmental stages of yak testis.

Rank	GeNorm		NormFinder		Best Keeper		Δ*Ct*		RefFinder	
1	*RPS15*	0.153	*TBP*	0.345	*UXT*	0.31	*TBP*	*0.82*	*TBP*	2.74
2	*RPL13A*	0.153	*YWHAZ*	0.460	*ACTB*	0.43	*YWHAZ*	0.87	*UXT*	3.46
3	*ACTB*	0.343	*HPRT1*	0.479	*GAPDH*	0.48	*HPRT1*	0.88	*YWHAZ*	3.87
4	*UXT*	0.386	*SDHA*	0.509	*RPL13A*	0.51	*SDHA*	0.94	*RPL13A*	4.47
5	*GAPDH*	0.485	*PPIA*	0.671	*SDHA*	0.52	*HMBS*	0.96	*SDHA*	4.68
6	*SDHA*	0.581	*UXT*	0.681	*RPS15*	0.54	*UXT*	0.97	*ACTB*	4.70
7	*TBP*	0.739	*HMBS*	0.684	*YWHAZ*	0.69	*PPIA*	1.01	*RPS15*	5.19
8	*YWHAZ*	0.816	*GAPDH*	0.855	*TBP*	0.72	*GAPDH*	1.08	*HPRT1*	5.20
9	*HPRT1*	0.857	*ACTB*	0.903	*HPRT1*	0.76	*ACTB*	1.09	*GAPDH*	5.57
10	*PPIA*	0.898	*RPL13A*	1.008	*PPIA*	0.77	*RPL13A*	1.13	*PPIA*	7.69
11	*HMBS*	0.923	*RPS15*	1.014	*HMBS*	0.94	*RPS15*	1.14	*HMBS*	8.07
12	*MRPL39*	0.975	*MRPL39*	1.044	*MRPL39*	1.19	*MRPL39*	1.17	*MRPL39*	12.00
13	*PPP1R11*	1.033	*PPP1R11*	1.259	*PPP1R11*	1.36	*PPP1R11*	1.35	*PPP1R11*	13.00

**Table 3:** Stability of RGs in immature stages of yak testis.

Rank	GeNorm		NormFinder		BestKeeper		Δ*Ct*		RefFinder	
1	*TBP*	0.191	*TBP*	0.258	*UXT*	0.30	*TBP*	0.55	*TBP*	1.57
2	*HMBS*	0.191	*HMBS*	0.287	*ACTB*	0.32	*HMBS*	0.56	*HMBS*	2.45
3	*YWHAZ*	0.254	*YWHAZ*	0.395	*RPL13A*	0.34	*YWHAZ*	0.60	*YWHAZ*	3.41
4	*GAPDH*	0.300	*GAPDH*	0.42	*RPS15*	0.36	*GAPDH*	0.64	*UXT*	4.05
5	*PPP1R11*	0.346	*UXT*	0.446	*YWHAZ*	0.37	*HPRT1*	0.67	*GAPDH*	4.76
6	*SDHA*	0.379	*HPRT1*	0.455	*PPP1R11*	0.42	*UXT*	0.67	*PPP1R11*	6.44
7	*MRPL39*	0.410	*PPPA1R11*	0.510	*TBP*	0.42	*PPPA1R11*	0.69	*ACTB*	6.69
8	*HPRT1*	0.442	*MRPL39*	0.523	*GAPDH*	0.44	*MRPL39*	0.69	*HPRT1*	7.00
9	*UXT*	0.518	*SDHA*	0.528	*HMBS*	0.44	*SDHA*	0.70	*RPL13A*	7.95
10	*ACTB*	0.577	*ACTB*	0.587	*HPRT1*	0.48	*ACTB*	0.75	*SDHA*	8.55
11	*RPL13A*	0.624	*RPL13A*	0.656	*SDHA*	0.49	*RPL13A*	0.79	*MRPL39*	8.56
12	*RPS15*	0.655	*RPS15*	0.704	*MRPL39*	0.67	*RPS15*	0.83	*RPS15*	9.12
13	*PPIA*	0.697	*PPIA*	0.807	*PPIA*	0.78	*PPIA*	0.93	*PPIA*	13.00

**Table 4:** Stability of RGs in mature stages of yak testis.

Rank	GeNorm		NormFinder		BestKeeper		Δ*Ct*		RefFinder	
1	*PPIA*	0.139	*TBP*	0.102	*HPRT1*	0.16	*TBP*	0.29	*MRPL39*	2.11
2	*MRPL39*	0.139	*MRPL39*	0.129	*PPIA*	0.28	*MRPL39*	0.29	*TBP*	2.63
3	*RPS15*	0.167	*RPL13A*	0.144	*UXT*	0.28	*RPL13A*	0.30	*PPIA*	3.08
4	*RPL13A*	0.174	*RPS15*	0.167	*RPS15*	0.31	*RPS15*	0.30	*RPS15*	3.72
5	*HPRT1*	0.204	*ACTB*	0.230	*MRPL39*	0.32	*PPIA*	0.34	*HPRT1*	3.96
6	*TBP*	0.227	*PPIA*	0.248	*ACTB*	0.35	*ACTB*	0.35	*RPL13A*	3.98
7	*UXT*	0.250	*HPRT1*	0.252	*RPL13A*	0.36	*HPRT1*	0.35	*UXT*	6.12
8	*ACTB*	0.271	*HMBS*	0.286	*TBP*	0.38	*HMBS*	0.38	*ACTB*	6.16
9	*HMBS*	0.290	*YWHAZ*	0.300	*HMBS*	0.40	*YWHAZ*	0.38	*HMBS*	8.49
10	*YWHAZ*	0.309	*UXT*	0.325	*PPPA1R11*	0.42	*UXT*	0.40	*YWHAZ*	9.93
11	*GAPDH*	0.327	*GAPDH*	0.331	*GAPDH*	0.44	*GAPDH*	0.41	*GAPDH*	11.00
12	*PPP1R11*	0.343	*PPP1R11*	0.374	*YWHAZ*	0.49	*PPP1R11*	0.44	*PPP1R11*	11.47
13	*SDHA*	0.360	*SDHA*	0.383	*SDHA*	0.56	*SDHA*	0.45	*SDHA*	13.00

## Supplementary Materials

The following are available online at https://www.mdpi.com/2076-2615/10/2/182/s1. Figure S1: Melting curves of 13 genes of RT-qPCR. Table S1: Primer-specific amplification efficiencies of the candidate RGs. Table S2: Averaged raw Ct values obtained for each candidate gene in all testis samples.
