# Supplementary material for: Validation of Suitable Reference Genes for Gene Expression Studies on Yak Testis Development
Source: Animals (Basel). 2020 Jan 21;10(2):182. doi: 10.3390/ani10020182 (PMC7070506; doi:10.3390/ani10020182)
Supplement: Supplementary file 1 [file animals-10-00182-s001.pdf]

**Table S1.** Primer-specific amplification efficiencies of the candidate reference gene.

| **Gene** | **Efficiency (%)** | **$R^2$** |
|---|---|---|
| ACTB | 92.0 | 0.9966 |
| GAPDH | 103.0 | 0.9948 |
| UXT | 108.0 | 0.9987 |
| TBP | 95.0 | 0.9972 |
| YWHAZ | 93.0 | 0.9999 |
| RPL13A | 92.0 | 0.9999 |
| SDHA | 105.0 | 0.9999 |
| RPS15 | 103.0 | 1.0000 |
| HPRT1 | 106.0 | 1.0000 |
| PPIA | 95.0 | 0.9900 |
| HMBS | 100.0 | 0.9999 |
| MRPL39 | 93.0 | 0.9946 |
| PPP1R11 | 102.0 | 0.9996 |
| TET2 | 104.0 | 0.9994 |

**Table S2.** Averaged raw Ct values obtained for each candidate gene in all testis samples.

| | **Sample Name** [1] | **PPIA** | **RPS15** | **MRPL39** | **PPP1R11** | **SDHA** | **TBP** | **UXT** | **ACTB** | **HMBS** | **GAPDH** | **YWHAZ** | **HPRT1** | **RPL13A** |
|---|---|---|---|---|---|---|---|---|---|---|---|---|---|---|
| | 6M4-1 | 20.29 | 19.28 | 26.27 | 27.26 | 28.59 | 27.45 | 24.63 | 19.42 | 31.08 | 28.05 | 23.33 | 27.68 | 21.92 |
| | 6M6-1 | 22.31 | 19.18 | 26.41 | 26.41 | 27.66 | 27.42 | 25.63 | 20.61 | 31.23 | 27.64 | 22.66 | 28.23 | 22.00 |
| | 6M7-2 | 22.41 | 19.08 | 26.47 | 26.85 | 27.27 | 27.49 | 25.48 | 20.56 | 30.81 | 27.65 | 22.69 | 28.12 | 21.92 |
| | 18M2-1 | 21.16 | 19.21 | 25.08 | 26.82 | 27.00 | 26.87 | 25.24 | 20.60 | 30.22 | 26.69 | 22.30 | 27.76 | 22.38 |
| | 18M4-1 | 22.43 | 19.82 | 26.27 | 26.38 | 27.87 | 27.30 | 25.88 | 20.73 | 30.80 | 27.13 | 22.47 | 28.25 | 22.69 |
| | 18M5-1 | 21.01 | 20.19 | 24.60 | 25.60 | 26.94 | 26.08 | 25.33 | 20.41 | 29.75 | 26.91 | 21.69 | 26.36 | 22.79 |
| | 30M10-1 | 20.05 | 20.20 | 23.22 | 23.92 | 26.77 | 25.67 | 25.61 | 21.23 | 28.89 | 27.59 | 21.10 | 26.12 | 23.09 |
| | 30M7-1 | 20.64 | 20.65 | 23.66 | 23.96 | 27.27 | 25.75 | 25.74 | 20.82 | 28.86 | 27.81 | 21.43 | 26.47 | 23.49 |
| | 30M8-1 | 20.29 | 20.33 | 23.69 | 24.39 | 28.08 | 26.19 | 25.46 | 21.34 | 28.69 | 28.22 | 21.76 | 26.41 | 23.17 |
| | 6Y-3 | 19.84 | 20.05 | 23.07 | 23.29 | 27.28 | 25.44 | 25.71 | 20.88 | 28.50 | 28.07 | 20.62 | 26.39 | 22.87 |
| | 6Y-4 | 20.08 | 20.35 | 23.20 | 23.12 | 27.17 | 25.47 | 25.93 | 20.97 | 28.28 | 27.86 | 20.57 | 26.38 | 23.03 |
| | 6Y-6 | 20.74 | 21.23 | 24.07 | 24.32 | 28.67 | 26.69 | 26.57 | 22.20 | 30.07 | 29.41 | 22.04 | 26.92 | 24.22 |
| All development stages | average | 20.94 | 19.96 | 24.67 | 25.19 | 27.55 | 26.49 | 25.60 | 20.81 | 29.76 | 27.75 | 21.89 | 27.09 | 22.80 |
| | SD | 0.91 | 0.64 | 1.31 | 1.45 | 0.60 | 0.78 | 0.44 | 0.62 | 1.04 | 0.67 | 0.82 | 0.81 | 0.66 |
| Immature stages | average | 21.60 | 19.46 | 25.85 | 26.55 | 27.55 | 27.10 | 25.37 | 20.39 | 30.65 | 27.35 | 22.52 | 27.73 | 22.28 |
| | SD | 0.83 | 0.40 | 0.73 | 0.52 | 0.57 | 0.50 | 0.39 | 0.44 | 0.51 | 0.47 | 0.49 | 0.65 | 0.36 |
| Mature stages | average | 20.27 | 20.47 | 23.49 | 23.83 | 27.54 | 25.87 | 25.84 | 21.24 | 28.88 | 28.16 | 21.25 | 26.45 | 23.31 |
| | SD | 0.32 | 0.39 | 0.35 | 0.48 | 0.64 | 0.44 | 0.36 | 0.47 | 0.57 | 0.59 | 0.55 | 0.24 | 0.45 |

[1] Sample Name: 6M-6 months, 18M-18 months, 30M-30months, 6Y-6 years.

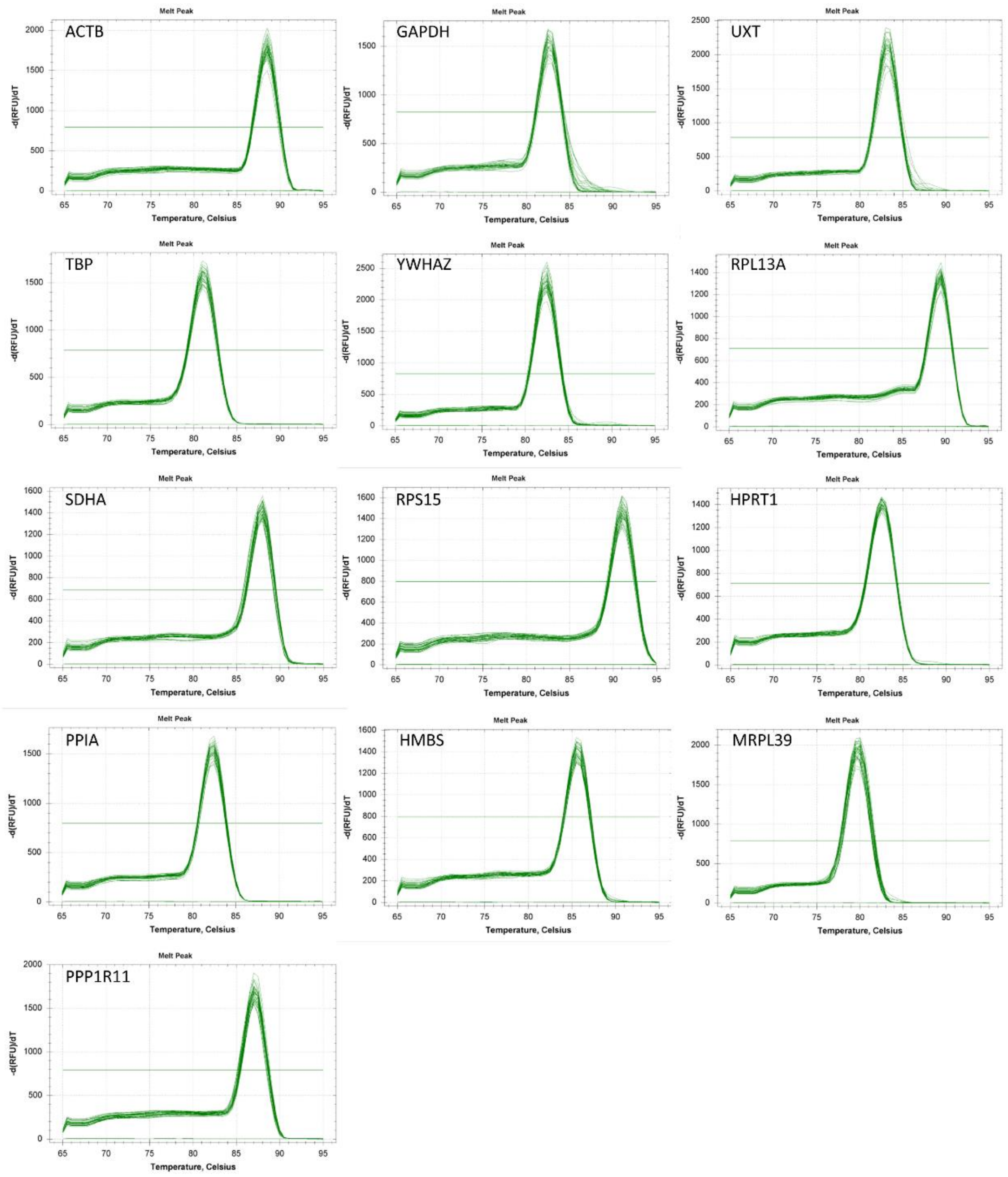


**Figure S1.** Melting curves of 13 genes of RT-qPCR.
